# Reticulate evolution in the *Pteris fauriei* group (Pteridaceae)

**DOI:** 10.1038/s41598-022-11390-7

**Published:** 2022-06-01

**Authors:** Yi-Shan Chao, Atsushi Ebihara, Wen-Liang Chiou, Jer-Min Tsai, Yu-Wen Huang, Tom A. Ranker

**Affiliations:** 1 Department of Biomedical Science and Environmental Biology, Kaohsiung Medical University, 100, Shih-Chuan 1st Rd., Kaohsiung, 80708 Taiwan; 2 Department of Botany, National Museum of Nature and Science, 4-1-1, Amakubo, Tsukuba-shi, Ibaraki, 305-0005 Japan; 3 Taiwan Forestry Research Institute, 53 Nan-Hai Rd., Taipei, 100051 Taiwan; 4 Department of Information and Communication, Kun Shan University, 195, Kunda Rd., Tainan, 710303 Taiwan; 5 University of Hawai‘i at Mānoa, School of Life Sciences, Honolulu, HI 96822 USA

**Keywords:** Evolution, Plant evolution

## Abstract

The *Pteris fauriei* group (Pteridaceae) has a wide distribution in Eastern Asia and includes 18 species with similar but varied morphology. We collected more than 300 specimens of the *P. fauriei* group and determined ploidy by flow cytometry and inferred phylogenies by molecular analyses of chloroplast and nuclear DNA markers. Our results reveal a complicated reticulate evolution, consisting of seven parental taxa and 58 hybrids. The large number of hybrid taxa have added significant morphological complexity to the group leading to difficult taxonomic issues. The hybrids generally had broader ranges and more populations than their parental taxa. Genetic combination of different pairs of parental species created divergent phenotypes of hybrids, exhibited by both morphological characteristics and ecological fidelities. Niche novelty could facilitate hybrid speciation. Apogamy is common in this group and potentially contributes to the sustainability of the whole group. We propose that frequent hybridizations among members of the *P. fauriei* group generate and maintain genetic diversity, via novel genetic combinations, niche differentiation, and apogamy.

## Introduction

Hybridization instantly creates novel combinations of genes and genomes^1^ and, therefore, can lead to rapid species differentiation^2–5^. Reticulate evolution of ferns was first reported in *Asplenium* species^6^. Subsequently, a growing number of studies have documented reticulate evolution in ferns, such as in *Dryopteris*, *Pteris*, and *Vandenboschia*^7–10^. However, the role of reticulate evolution in promoting diversification remains unclear. For example, with various phenotypes arising quickly, hybridization may promote adaptive radiation^3,11–13^. In angiosperms, rapid diversification is driven by various morphological, physiological, and genetic characters, as well as the origin of physical barriers that may combine to promote different ecological fidelities between parental and hybrid species^14–18^.

Hybrids may spread to new ecological habitats, differing from those of the parental species, thus reducing competition with parental species and potentially facilitating hybrid speciation^19,20^. A long-term field experiment of *Helianthus* provided direct evidence of hybridization driving adaptive radiation ^21^. In other words, ecological divergence of hybrids is likely to be related to the success of hybrid speciation ^22–25^, but the role of niche differentiation in the evolution of hybrid species in ferns has been little studied^26–28^. Ecological differentiation in hybrid zones was first proposed in the *Pteris quadriaurita* complex^7,29^, and then reported in *Polystichum imbricans* (D.C.Eaton) D.H.Wagner and *Polystichum munitum* (Kaulf.) C.Presl (Dryopteridaceae)^30^.

In ferns, hybridization is often accompanied by apogamy^31,32^. Apogamy is found in 3% to 10% of fern species^33,34^ and results from the deregulation of reproductive pathways^35^. Apogamous hybrid ferns are reported in many cryptic complexes, especially in Aspleniaceae^36,37^, Dryopteridaceae^38–40^, and Pteridaceae^9,28,41^. Because apogamous species often have limited genetic variation compared to close sexual relatives ^42^, apogamy has been considered a dead end for fern evolution^43^; however, it is common and could play a special role in *Pteris*^29,44,45^, where 21% of species reproduce only by apogamy and 8% of species have both sexual and apogamous individuals. Apogamous gametophytes could produce functional male gametes and serve as paternal parents when crossed with gametophytes of allied sexual species^29,46,47^. Similar scenarios have been proposed in the *Pteris cretica*, *Dryopteris varia*, and *Diplazium hachijoense* complexes^48–50^.

Interestingly, apogamous *Pteris* species are most prevalent in East Asia and South Asia and mostly found in sect. *Campteria*^45^. The *Pteris fauriei* group (Pteridaceae), belonging to sect. *Campteria*, includes more than 20 taxa (18 species) with bipinnatifid laminae^51^. Apogamy of many taxa in this group is probably associated with hybridization^51,52^. In addition, similar morphology among species in this group is thought to be due to hybridization. For example, Kuo^53^ suggested that *Pteris wulaiensis* C. M. Kuo was a hybrid between *P. faurei* Hieron. and *P. bella* Tagawa. The *Pteris fauriei* group is mainly distributed in Eastern Asia and taxa have diverse geographic distributions and niche fidelities. *Pteris fauriei*, *P. arisanensis* Tagawa, *P. biaurita* L., and *P. latipinna* Y.S.Chao & W.L.Chiou are common and distributed widely^51,54^, but most taxa in the *P. fauriei* group are endemic to small regions. For example, *P. boninensis* H.Ohba, *P. laurisilvicola* Sa.Kurata, *P. natiensis* Tagawa, *P. satsumana* Sa.Kurata, and *P. yakuinsularis* Sa. Kurata are limited to Japan^55^, *P. confusa* T.G.Walker is found in India and Sri Lanka^56^, *P. austrotaiwanensis* Y.S.Chao is only reported in south Taiwan^50^, and *P. kawabatae* Sa.Kurata, *P. minor* (Hieron.) Y.S.Chao, and *P. wulaiensis* are recorded in Japan and Taiwan^51,57^. In regards to niche diversity, most taxa occur in shady environments, like *P. latipinna*, or semi-open habitats, like *P. fauriei*, although *P. minor* occurs in open habitats^51,58^.

We addressed the following questions of the *P. fauriei* group: (1) Are any species of the *P. fauriei* group of hybrid origin? Chloroplast and nuclear DNA data were analyzed phylogenetically and integrated with ploidy level data to infer a possible network of hybridization. (2) Is morphological diversity related to hybridization? Morphological characteristics and genetic composition of each taxon were compared. (3) Is niche diversity related to hybridization? General attributes of ecological niches were compared among taxa. (4) What is the role of apogamy in the evolution of the *P. fauriei* group? Contributions and/or disadvantages of apogamy in hybridization were also discussed.

## Results

### Morphological and habitat characters

Samples of the *Pteris fauriei* group were collected from Eastern Asia, including Japan, Taiwan, China, Vietnam, and Malaysia, and included 20 taxa (18 species; Supplementary Table 1). No material of *P. kiuschiuensis* var. *centrochinensis* was collected for molecular analysis, and *P. confusa* and *P. oshimensis* var. *paraemeiensis* were amplified only for cpDNA markers. Some sampled plants morphologically matched the type specimens (Table 1); other plants were attributed to the most morphologically similar species. We found that the taxa had different niche attributes, such as light intensity and elevation (Table 1). All species occurred in shady or semi-shady environments, except *P. minor*, which was found in open and sunny areas. Most taxa grew at low elevations (≤ 1000 m), but *P. arisanensis*, *P. biaurita*, and *P. setulosocostulata* were located at both low and higher elevations (> 1000 m).

**Table 1:** The ploidy levels, cpDNA haplotypes, nDNA genotype, inferred maternal lineage, paternal lineage, and geographic distribution of samples in the *Pteris fauriei* group. Plants having identical morphology with the type specimens are indicated as Y.

Scientific name (Morphology-based)	Type	Hybrid formula (♀ × ♂)†	*Knox3*	*IBR3*	cpDNA haplotype	Ploidy	Reproductive mode	Maternal lineage of *Knox3*	Maternal lineage of *IBR3*	Samples (one representative)	Distribution; elevation (≤ 1000, l; > 1000, h)	Ploidy data of previous studies
*P. arisanensis*	Y	–	F7F7F7	Y21Y87*	cxx	3X	apo	F7	Y87	Chao2135	Taiwan; h	3X ^61^
	Y	–	F7F23	Y21Y87	cxx	–	apo	F7	Y87	Yang191030002	Taiwan; h	
		–	F4F5	Y21V22	cx	2X	apo	F4 or F5	Y21	Chao2482	Taiwan; l	
		*–*	A5D4G1	S19T20*	cf'	3X	apo			Chao2296	Taiwan; l	
		*P. latipinna* × *P. arisanensis*	D7F4F5	Y21V22W23	ca	3X	apo	D7	W23	Hsu s.n.20130116	Taiwan; h to l	
		*–*	D5F3	W23Y24	ca	2X	apo	D5	W23	Chao2483	China, Taiwan, Vietnam; l	
		–	D7F3G1	W5S14Y24	ca	3X	apo	D7	W5	Chao2867	China; l	
		–	=	S14V22W23	ca	3X	apo	D7	W23	Chao2858	China; l	
*P. austrotaiwanensis*		–	D3D5	W69W23	ct	2X	apo	D3	W69	Hsu s.n.20130315	Taiwan; l	
*P. biaurita*	Y	–	–	S25S25	ci	–		G1	S25	Kao 03,037	Costa Rica; l	
			G1G1	S14S14	ci	2X	apo	G1	S14	Chao2734	Cambodia, Thailand, China; l	
		–	G1G4	S19S19	ci	2X	apo	G1	S19	Chao2419	Malaysia; l	
		–	G1G3	S19S19	ci	–	apo	G1	S19	Chao2752	China, Thailand; l	
		*P. biaurita* × *P. arisanensis*	F7G1	S14S19Y21	ci	–	apo	G1	S14 or S19	Chao1317	China; h	
		=	F7G1*	S14Y21S25	ci	3X	apo	G1	S14 or S25	Chao2484	Taiwan; h to l	
		=	=	Y21S70*	ci	3X	apo	G1	S70	Chao2471	Taiwan; h to l	
		*P. arisanensis* × *P. biaurita*	F16G1	S14Y87	cxx	2X	apo	F16	Y87	Chao2478	Taiwan; h to l	
		=	=	=		–	apo	G1	S14	Yang191029002	Taiwan; h	
		*P. latipinna* × *P. biaurita*	D7G1	S25W23	ca’	2x	apo	D7	W23	Chao2869	China; l	
*P. boninensis*	Y	*–*	A7A7	T1T1	cb	2X	sex	A7	T1	Chao1942	Japan; l	2X ^62^
		*P. boninensis* × unknown	A1A7D5	W2T1*	cb	3X	apo	A7	T1	Chao1818	Japan; l	
		=	=	W2T1T7	cb	3X	apo	A7	T1	Chao1819	Japan; l	
*P. confusa*	Y	*–*	–	–	cc	–	apo	-	–	Lu32448	Sri Lanka; l	
*P.* aff. *confusa*			F1F1	Y73Y73		–	apo	F1	Y73	CRFJ FN 402	Nepal; l	
			F1F2	–		–	apo	F1	Y73	CRFJ 34,934	India; l	3X ^63^
*P. fauriei*	Y	*P. minor* × *P. latipinna*	A1A6D7	W5T1*	cf	3X	apo	A1 or A6	T1	Chao2082	Japan, Taiwan; l	
	Y	=	A1D7*	W5T1*	cf	3X	apo	A1	T1	Yang191005005	Taiwan; l	
	Y	=	A6D7	W5T1	cf	2X	apo	A6	T1	Chao2668	Taiwan; l	
	Y	=	A6D7*	W5T1*	cf	3X	apo	A6	T1	Chao2667	China, Japan, Taiwan; l	
		–	A3A5D7	W5T1*	cf	3X	apo	A3 or A5	T1	Chao2051	Japan; l	
		*–*	A11D32*	W5T1*	cf	3X	apo	A?	T1	Chao2805	Taiwan; l	
		*P. minor* × *P. latipinna*	A1A13D7	W5T1	cf	–	apo	A1	T1	Chao2790	Taiwan; l	
*P.* cf. *fauriei*		*P. oshimensis var. oshimensis* × *P. latipinna*	A4D7	W5T1	cf	2X	apo	A4	T1	Chao2155	Taiwan; l	
		=	A4D7*	T1T4W23	cf	3X	apo	A4	T1	Chao2156	Taiwan; l	
		–	A5A12D8	W5T1T4	cf	3X	apo	A5 or A12	T1 or T4	Wade3659–1	Japan; l	
		–	C1D1	V12X79	cs	2X	apo	C1	X79	Chao2028	Japan; l	
		*P. wulaiensis* × *P. latipinna*	D4D7	W28W23	cy	2X	apo	D4	W28	Chao2035	Japan, Taiwan; l	
		=	=	W29W23	cy	2X	apo	D4	W29	Chao2553	Taiwan; l	
		=	=	W5W28	cy	–	apo	D4	W28	Lu25409	China; l	
*P. kawabatae*	Y	–	C3E2	V41V86	ce	–	apo	E2	V86	Lu22877	Taiwan; l	
		–	B4D2H4	S14W52X82	cs	––	apo	B4	X82	Chao2170	China; l	
		–	B4D5H1	W23X82Y11	cs	3X	apo	B4	X82	Lu28259	Taiwan; l	
		–	B4H3	X82Y11	cs	2X	apo	B4	X82	Lu28430B	Taiwan; l	
		–	C3D7E2	W23V41V86	ca	–	apo	D7	W23	Knapp 4145	Taiwan; l	
		–	C3D1	V12W75	cl	2X	apo	D1	W75	Chao2022	Japan; l	2X, Ebihara 3239 from the same population with Chao2022, ID as *P. laurisilvicola* ^52^
*P. kiuschiuensis*	Y	–	C3H1	V12Y11	ck	2X	apo	H1	Y11	Chao1852	China, Japan; l	2X ^64,65^
		–	C3D4H1	W5W78Y11	cy	3X	apo	D4	W78	Ebihara et al. 3240	Japan; l	3X, ID as *P. yakuinsularia* ^52^
		–	=	W78V12Y11	cy	3X	apo	D4	W78	Lu24743	China, Japan; l	
		–	D4D7H1	V12Y11W29	ck	–	apo	H1 = H6	Y11	Chao2182	China; l	
*P. latipinna*	Y		D7D7	W5W5	ca	2X	apo	D7	W5	Chao2092	China, Taiwan; l	
			=	W2W2	ca	–	apo	D7	W2	ZXC001673	Taiwan; l	
*P. laurisilvicola*	Y	–	A4A5D7	W30T1T4	cf	3X	apo	A4	T1	Chao2555	Japan, Taiwan; l	
		–	A4A6D7	W5T1T4	cf	3X	apo	A4 or A6	T1	Chao2528	Taiwan; l	
		–	=	W30T1T4	cf	3X	apo	A4 or A6	T1	Chao2891	Taiwan; l	
*P. minor*	Y		A1A1	T1T1	cf	2X	sex	A1	T1	Chao2078	Taiwan; l	
	Y	–	A1A13	T1T1	cf	2X	sex	A1	T1	Chao2647	Taiwan; l	
	Y		A1A6	T31T31	cf'	2X	sex	A1 or A6	T31	Chao2500	Taiwan; l	
	Y		A6A6	T1T1	cf	2X	sex	A6	T1	Hsu 8425	Taiwan; l	
	Y		=	T27T27	cf'	2X	sex	A6	T27	Chao2653	Taiwan; l	
*P. natiensis*	Y	unknown × *P. latipinna*	C3D7	V12W23	cn	2X	apo	C3	V12	Chao1842	Japan; l	2X ^64^
		–	C3D4D7	V12W28W23	cy	3X	apo	D4	W28	Chao2017	China, Japan; l	
*P. oshimensis* var. *oshimensis*		var*. oshimensis* × unknown	A4D1	T1W75	cf	2X	apo	A4	T1	Chao1881	Japan; l	2X, Ebihara 3239 from the same population with Chao1881^52^
	Y		A4A4	T1T1	cf	2X	sex	A4	T1	Kuo3445	Japan; l	2X, Ebihara 3379 from the same population with Kuo3445 ^52^
*P. oshimensis* var. *paraemeiensis*	Y	*–*	–	–	cs	–	apo	–	–	Zhang et al.20100430109	China; l	
*P. pseudowulaiensis*		–	D1D5	W5W52	cw	–	apo	D5	W5	Hsu 8437	Taiwan; l	2X ^46^
		–	D2D5	W52W23	cw	2X	apo	D5	W23	Ko33986	China; l	
		*P. wulaiensis* × unknown	D4D5	W5W28	cy	2X	apo	D4	W28	Wade2315	China, Taiwan; l	
*P. satsumana*	Y	–	B1C3	V12X82	cs	2X	apo	B1	X82	Chao1925	Japan; l	2X ^52^
*P. setulosocostulata*	Y	–	D4H4H10	W52Y11W89	ch	3X	apo	H4	Y11	Chao2526	Taiwan; l	3X ^61,66^
		–	B2H3H5	X82Y11	ch	–	apo	H3	Y11	Chao1363	China; l	
*P. wulaiensis*	Y	–	D2D4	W52W75	cd	2X	apo	D2	W52	Hsu9088	Taiwan; l	
		–	D4D4	W78W78	cy	2X	apo	D4	W75	Ebihara et al. 3234	Japan; l	2X, ID as *P. oshimensis* ^52^

– No data. = The same as the above field. * Unasserted alleles. †Parents in the inferred hybrid formula are based on the species (or taxa) with homozygous genotypes.

### Ploidy levels and reproductive modes

There were diploids and triploids in the *P. fauriei* group based on flow cytometry data; some named taxa had both diploids and triploids. Previous studies on ploidy levels in the *P. fauriei* group are cited in Table 1. There were seven taxa with homozygous nDNA genotypes that could be potential parental taxa (inferred by nDNA described in the section below): *P. arisanensis*, *P. biaurita*, *P. boninensis*, *P. latipinna*, *P. minor*, *P. oshimensis* var. *oshimensis*, and *P. wulaiensis*. These are all diploids except for *P. arisanensis,* which has some homozygous triploid individuals. Only three of the diploid taxa—*P. boninensis*, *P. minor*, and *P. oshimensis* var. *oshimensis—*had 64 spores per sporangium, which indicated that they reproduce sexually. The other plants had 32 spores per sporangium, which we inferred to be apogamous (Table 1; Supplementary Table 1). It has been reported that sexual *Pteris* species produce 64 spores per sporangium whereas apogamous species produce 32 spores per sporangium, especial in *P. fauriei* and *P. minor*^59^. Each plant produced only one type of sporangium.

### The phylogeny of chloroplast DNA and nuclear DNA

**Figure 1:**
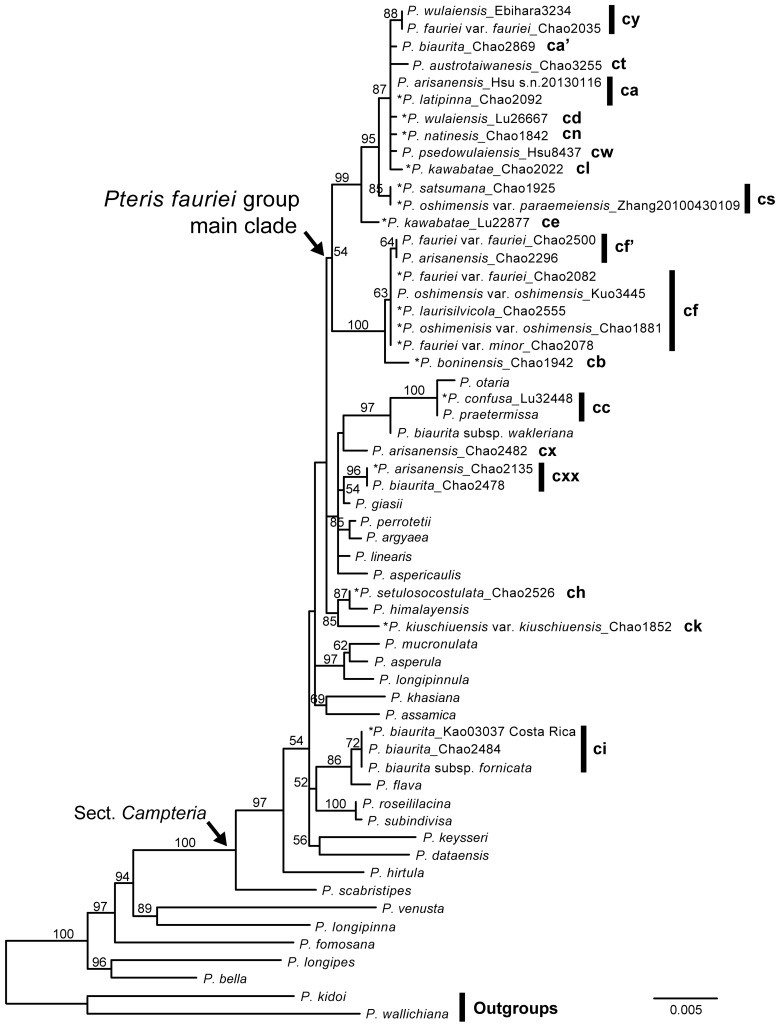
Chloroplast DNA phylogeny of haplotypes of *Pteris fauriei* group. ML bootstrap support values are indicated on each branch. * indicates the samples with the greatest morphological similarity to corresponded type specimens.

The sequences of *rbcL, matK*, *IBR3*, and *Knox3* of the *P. fauriei* group in this study (Supplementary Table 1) were clustered and named as haplotypes and allele types. Phylogenetic statistics are shown in Table 2. There were 20 cpDNA haplotypes; some were specific to one species and some were shared by several species. We included different taxa, different species and/or one species with different cpDNA haplotypes, of the *P. fauriei* group in the cpDNA phylogenetic analyses. For each species, samples with different haplotypes were included, and the samples with morphology identical to type specimens were marked (Fig. 1). The cpDNA topology was similar to the previously published *Pteris* phylogeny ^60^, which resolved the *P. fauriei* group as part of sect. *Campteria*, although the supporting value was not very high.

**Table 2:** The characters of DNA datasets of *Pteris fauriei* group.

Dataset	Number of sequences	Haplotypes/ alleles numbers	Total characters	Parsimony-informative characters	Log-likelihood score for ML tree
*rbcL* + *matK*	57	19	2184	165	−6583.6080
*Knox3*	46	38	493	109	−2096.8907
*IBR3*	39	34	397	45	−1268.7633

Taxa belonging to the *P. fauriei* group are coded based on their haplotypes, such as ca, cc, and cf. Most species clustered within the main *fauriei* clade, but *P. confusa* (cc), *P.* aff. *confusa* (cu), *P. kiuschiuensis* (ck), and *P. setulosocostulata* (ch), as well as some plants of *P. arisanensis* (cx, cxx) and *P. biaurita* (ci, cxx) had distant phylogenetic positions outside of the main clade. Only *P. austrotaiwanensis* (ct), *P. boninensis* (cb), *P. confusa* (cc), *P. kiuschiuensis* (ck), *P. natiensis* (cn), *P. pseudowulaiensis* (cw), and *P. setulosocostulata* (ch) had their own unique cpDNA haplotypes (Table 1, Fig. 1); most taxa had more than one cpDNA haplotype. Some taxa shared haplotypes, indicating shared maternal lineages, such as *P. arisanensis* and *P. biaurita* (cxx), *P. arisanensis* and *P. latipinna* (ca); *P. fauriei*, *P. minor*, *P. laurisilvicola*, and *P. oshimensis* var. *oshimensis* (cf); *P. oshimensis* var. *paraemeiensis* and *P. satsumana* (cs); and *P. wulaiensis* and *P. fauriei* (cy). The two varieties of *P. oshimensis* have different cpDNA haplotypes. Based on this fact, together with their distinctly different morphologies, we suggest that *P. oshimensis* var. *paraemeiensis* is a new species awaiting taxonomic revision.

**Figure 2:**
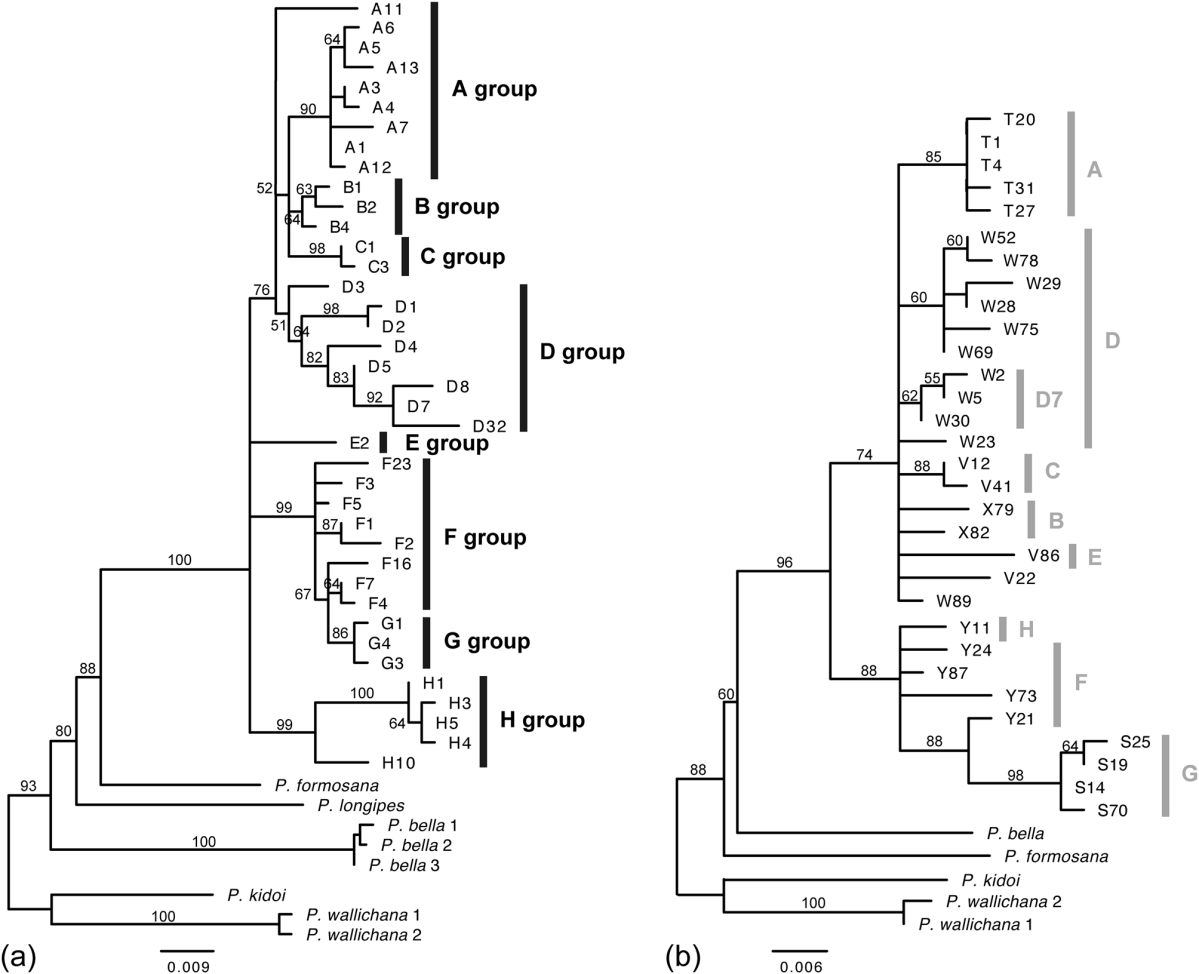
Nuclear DNA phylogeny of the *Knox3* gene (**a**) and the *IBR3* gene (**b**) of the *Pteris fauriei* group. ML bootstrap support values are indicated on each branch.

**Figure 3:**
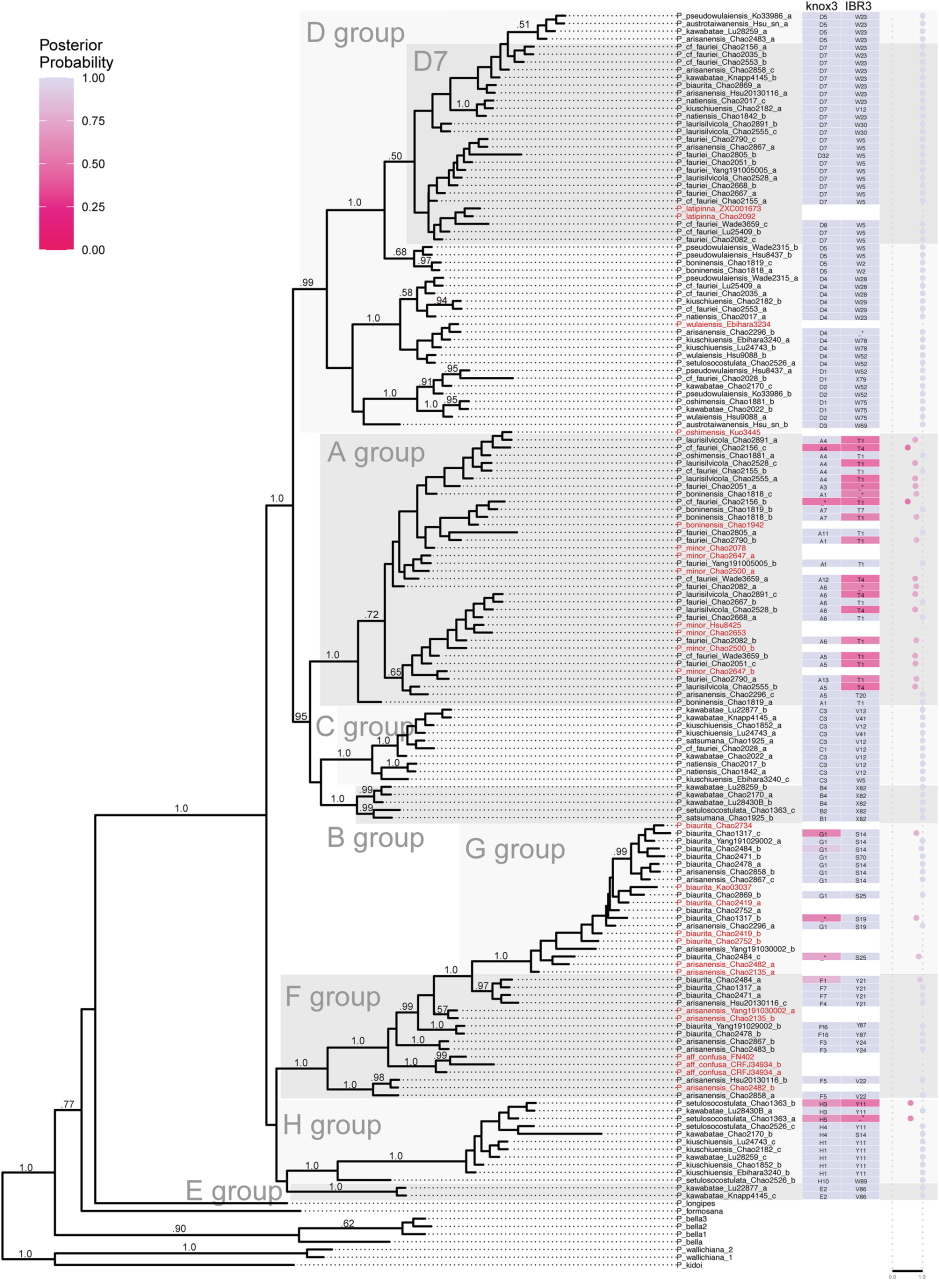
The phasing inference of the *Knox 3* gene and the *IBR3* gene of the *Pteris fauriei* group by homologizer^75^. Bayesian posterior probabilities of branches are indicated, and the values < 0.5 are not shown. The two heatmap columns show the corresponding alleles of the two nDNA genes and are colored by the marginal posterior probability of the phase assignment. Another column presents the mean marginal probability across the two loci of the phasing assignment per tip. Taxa in red are putative parents. All the samples of the *Pteris fauriei* group are the same as those listed in Table 1.

The phylogenetic analysis of the *Knox3* alleles supported six clades, which we labeled B, C, D, E, F + G, and H (Fig. 2a.). Group A is related to *P. minor*, and groups F and G correspond to *P. arisanensis* and *P. biaurita*, respectively, and are interdigitated. The *IBR3* topology (Fig. 2b) approximately corresponded to that based on *Knox3*. Together with ploidy data, the genotypes of *IBR3* and *Knox3* were determined (Supplementary Table 1). Most of the genotypes were found in two or more samples from each taxon, except *P. kawabatae*, in which each individual sampled had a unique genotype (Table 1). The *IBR3* gene exhibited fewer alleles than the *Knox3* gene (Table 1) and had fewer genotypes, labeled as S, T, V, W, X, and Y. For example, samples with *Knox3* genotype A1A1 and A4A4 were both *IBR3* genotype T1T1 (Table 1). In some taxa, however, *IBR3* exhibited more variation than *Knox3*, such as samples with *Knox3* genotype F7G1* showing five *IBR3* genotypes (Table 1). We also phased the alleles of the two nDNA genes, to infer subgenome evolution of this group (Fig. 3). There is a mean posterior probability of − 3437.35, and the effective sample size (ESS) was 292. The results support that the two nDNA genes present similar evolutionary histories, and the inferred phylogeny is generally consistent with the pattern of two DNA phylogenies in Fig. 2. Potential parental taxa with homozygous nDNA genotypes were shown empty in the nDNA columns. Most of the posterior probabilities of the phase assignments were high (heatmap in blue), and potential missing data (not be amplified alleles) are shown as having low posterior probability (heatmap in red).

**Figure 4:**
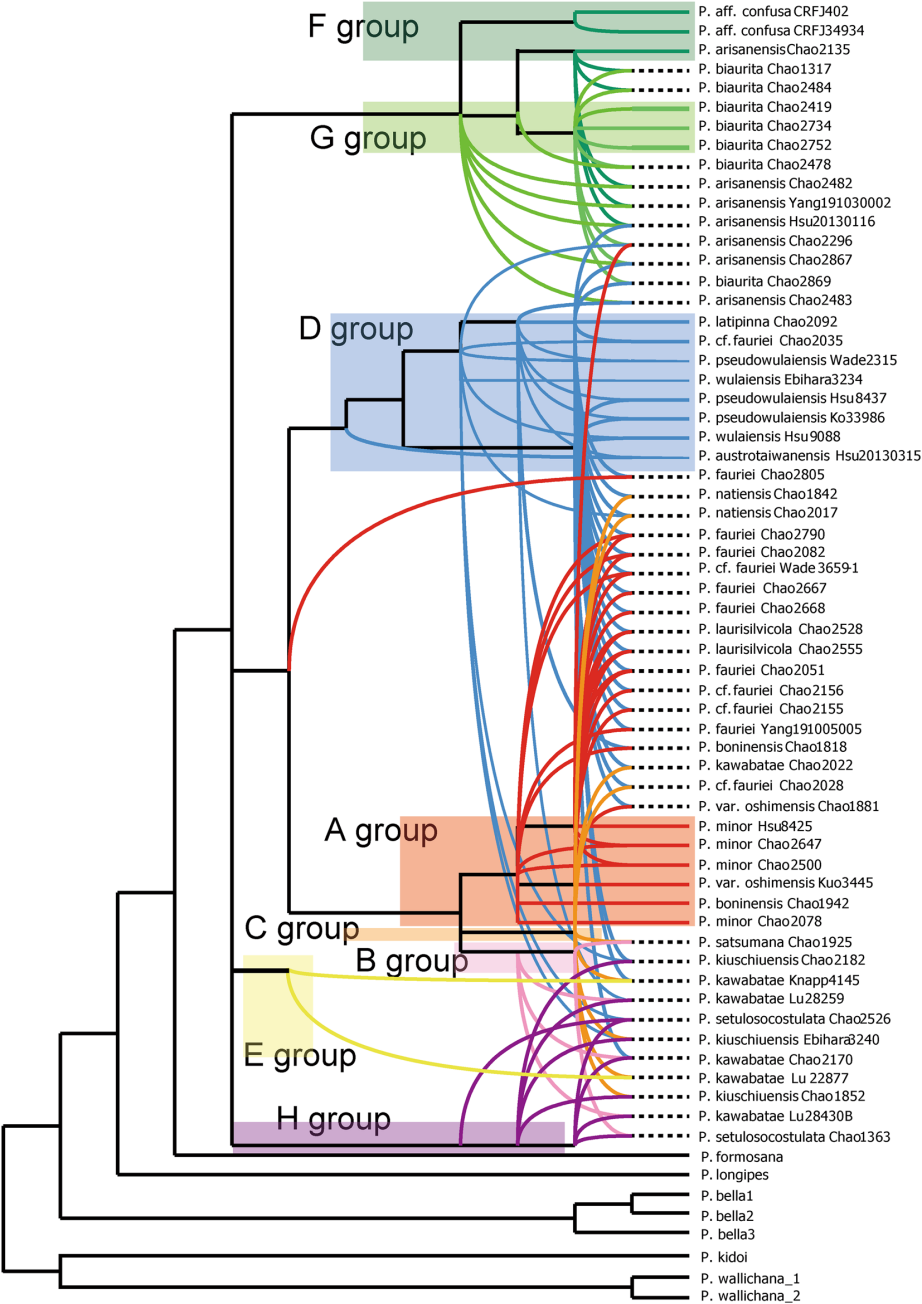
Phylogenetic network of the *Pteris fauriei* group, based on *Knox3* gene. Color of line indicates lineage of *Knox3* grouping. A group in red; B group in light purple; C group in orange; D group in yellow green; E group, in blue; F group in green; G group in limon. The dash black lines mean hybrid taxa. The taxa correspond to samples in Table 1.

The following analyses were based on the *Knox3* gene results mainly because of their higher resolution than the *IBR3* gene and show clearer lineages than the phasing patterns to infer hybridization relationships between potential paternal taxa and hybrids. Subsequently, a phylogenetic network was inferred based on *Knox3* alleles and genotypes (Fig. 4). The crossing patterns (taxa in Table 1) were inferred. Lineages of A to G groups were colored separately, and then the lineages of putative hybrid taxa were indicated. The topology presents an evolutionary radiation involving hybridization; many hybrid taxa arose from only a few parental lineages.

### Parent and hybrid taxa assignment

**Figure 5:**
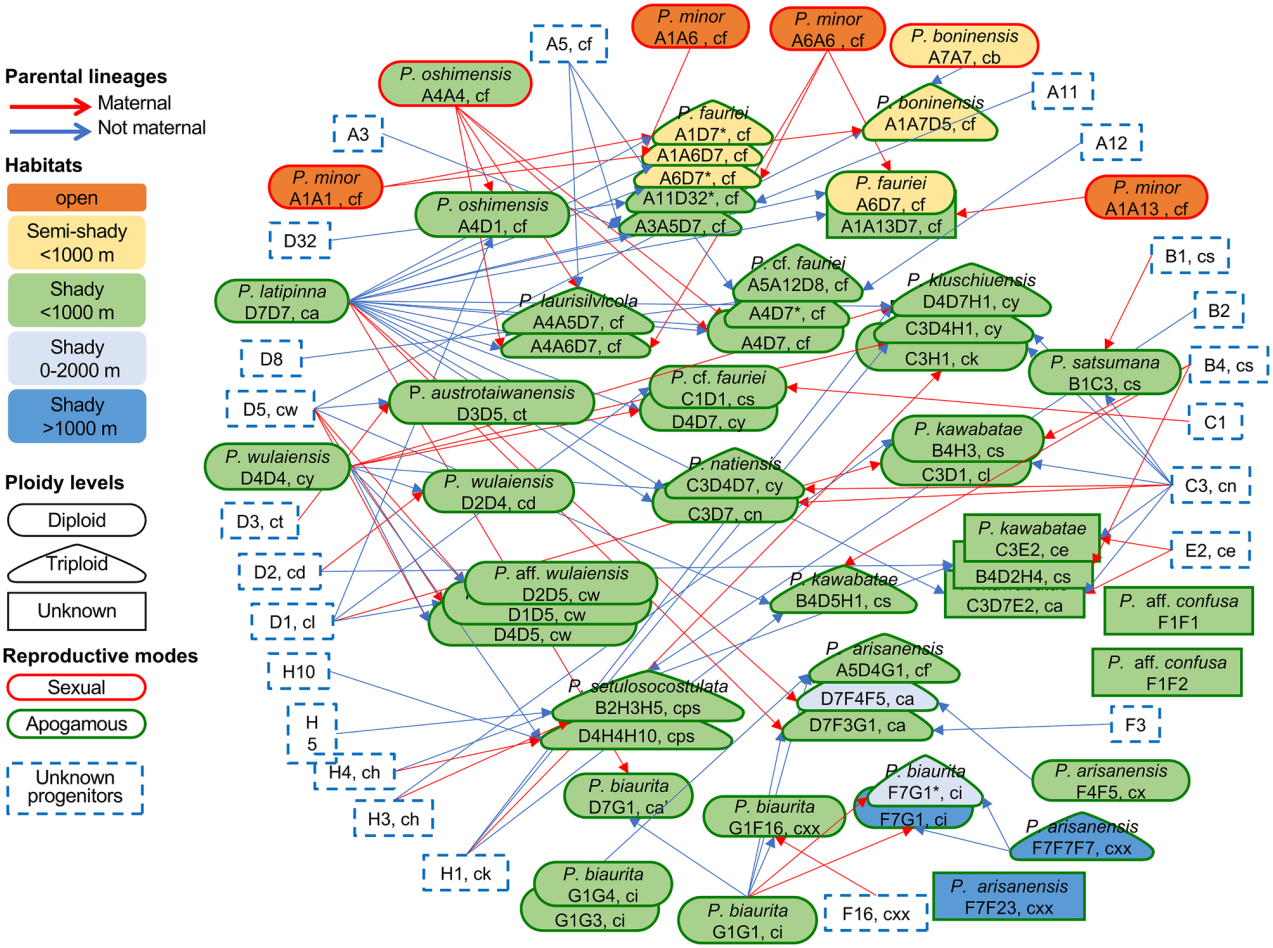
A reticulogram of the *Pteris fauriei* group, based on the *Knox3* sequences. The taxa correspond to the genotypes in Table 1. Maternal contributors of hybrids are shown as red arrows and paternal contributors as blue arrows. Ploidy levels are indicated by symbols: ellipse for diploids, triangle for triploids, and rectangle for unknown ploidy. Different kind of habitats are separated by colors inside symbols. Red or green symbol outlines indicate reproductive mode (sex and apogamy, respectively). Undiscovered taxa have dashed-lined outlines. Alleles of *Knox3* and cpDNA haplotypes of each taxon are also presented.

By comparing cpDNA haplotypes (Fig. 1) and the phasing of nDNA genotypes (Fig. 3), the maternal lineages of the nDNA genotypes were inferred (Table 1). For example, the cf, cf’, and cb cpDNA haplotypes were always present along with the group A allele of nDNA, and ca of cpDNA was found with allele D7 of *Knox3* nDNA. A reticulogram based on the *Knox3* gene was constructed, onto which we mapped ploidy levels, habitats, and reproductive modes (Fig. 5); the taxa correspond to the taxa in Table 1. Sexual plants were few, so we assumed that the probabilities of backcrossing with parents and introgression were low, especially with the apogamous diploid. Furthermore, the two nDNA markers presented similar topologies.

Based on the *Knox3* gene, 72 genotypes were inferred, including 14 putative parental genotypes and 58 genotypes of hybrid origin (Table 1). All species were identified based on morphology. Plants with homozygous genotypes or some sexual diploid genotypes possessing alleles from the same groups, are proposed as possible parental taxa. Seven putative parental taxa were discovered. Strictly speaking, only samples morphologically identical with type specimens of *P. arisanensis* (F group, F7F7F7), *P. boninensis* (A7A7), *P. minor* (A group except A7 & A4, A1A1, A1A13, A1A6, A6A6), and *P. latipinna* (D7D7) were homozygous, which suggests that they were not hybrid taxa. Most taxa in the *P. fauriei* group, however, appeared to be of hybrid origin. Although the other three putative species had homozygous samples, such as *P. biaurita* belonging to group G (samples with G1G1), *P. oshimensis* var. *oshimensis* belonging to A4 (Kuo 3445, A4A4), and *P. wulaiensis* belonging to D4 (Ebihara et al. 3234, D4D4), they also had heterozygous taxa. It was difficult to separate the homozygous samples and heterozygous samples based on their morphology.

*Pteris latipinna* (D7D7) was the putative parental lineage of 18 hybrid taxa (Fig. 4), far more than any other species. *Pteris wulaiensis* (D4D4) was the second most important parental lineage, contributing to eight taxa. Homozygous *P. oshimensis* var. *oshimensis* (A4A4) was inferred to be the maternal parent of hybrid *P. oshimensis* var. *oshimensis* (A4D1) and *P.* cf. *fauriei* (A4D7).

The *Knox3* alleles of F and G groups, corresponding to *P. arisanensis* and *P*. *biaurita*, respectively, were clustered together within one clade (Fig. 2). The two species can be identified by morphology (especially the costal veins, described in the section below) and were phylogenetically close based on *Knox3*. Some taxa were inferred to arise from hybridization of the two species, with each species serving as either paternal or maternal parent, such as F16G1 (*P. arisanensis* (♀) × *P. biaurita* (♂)) and F7G1 (*P*. *biaurita* (♀) × *P. arisanensis* (♂)). However, the possible parental individuals (F7F7F7 in *P. arisanensis*; G1G1, G1G4, G1G3 in *P. biaurita*) were all apogamous. We have not found sexual diploid individuals of the two species. *Pteris latipinna* was also involved in hybrid formation with each of those two species, such as D7F4F5 in *P. arisanensis* and D7G1 in *P. biaurita*.

Unique alleles only appeared in a few rare taxa. The C3 allele was found in *P. kawabatae* (C3D1, C3D7E2, C3E2), *P. kiuschiuensis* var. *kiuschiuensis* (C3H1, C3D4H1), *P. natiensis* (C3D7, C3D4D7), and *P. satsumana* (B1C3). Allele E2 was only found in *P. kawabatae* (C3D7E2, C3E2). Alleles H3, H5, and H10 were unique to *Pteris setulosocostulata* (D4H4H10, B2H3H5). Allele H1 was found in *P. kawabatae* (B4D5H1) and *P. kiuschiuensis* var. *kiuschiuensis* (C3H1, C3D4H1, D4D7H1). The relationships of *P. setulosocostulata* to *P. kawabatae* and *P. kiuschiuensis* var. *kiuschiuensis* were unclear because the sample sizes were small for the latter two taxa.

### Genotypes resolving puzzles of morphology and habitats

The diverse morphology of the *P. fauriei* group appeared to be an outcome of a large number of hybridizations. By comparing morphological characters^51^ and molecular data (Table 1), the association of specific morphological character states with particular genotypes was explored. Some character states were only found in individuals with particular alleles (Table 3). We present apparent associations between morphological and ecological variation and genotypic markers as hypotheses that could be tested with more detailed analyses. We found that hybrid taxa exhibited the morphology associated with their parents (Table 1).

**Table 3:** The genetic lineage inferences of morphological and habitat characters^51^, based on the *Knox*3 gene marker (Table 1) of *Pteris fauriei* group. The taxa of *Pteris fauriei* group exhibit the morphology corresponded to their own genotypes.

Characters	State 1	Alleles	State 2	Alleles	State 3	Alleles
1) Lamina ratio of length to width	1.1–1.3	A1, C3, D7	1.4–1.7	Undetermined, except A1, C3, D7	1.8–2	Undetermined, except A1, C3, D7
2) Stipe color	Stramineous or green	Except D4	Red-brown	D4		
3) Stipe base thick	2.5–4 mm	Except A4, D5	Stipes < 2.5 mm	A4, D5		
4) Exaggerated basiscopic pinnae	1 pair	Undetermined	2 or more pairs	A1, A4, A6, H1	Almost tripartite	A1, A4, A6
5) Number of lateral pinnae	< 6	C3, D4, D7	6–8	–	≥ 9	A4, H1
6) Pinna angles against rachis	60–70°	Undetermined, except C3	71–80°	Undetermined, except C3	81–90°	C3
7) Pinna straight or incurve	Straight	Except C3, H1	Incurved	C3, H1		
8) Pinnule width	> 3 mm	D4, D5	≤ 3 mm	Except D4, D5		
9) Pinna stalks	Distinct	D4	Sessile	Except C3, D4, H group	Basal segments connecting to midribs	C3, H group
10) Basal pinnules of lateral pinnae	Not decurrent, falcate	–	Decurrent, triangular	A4, A7, D7, F		
11) Pinnae apexes	Acute or caudate, short tails < 2.5 cm	–	Caudate, long tails > 3 cm	A7, C3, D7		
12) Pinna width	> 3 cm	A4, D4, H1	3–4 cm	–	> 4 cm	D7
13) Length ratio of basiscopic pinnules with acroscopic ones	1–1.4	–	1.5–2	A5, C3		
14) Pinna width variation	Equally wide	–	Narrowed at base	D4, D7	Widest at base	A4
15) Pinnule apexes of sterile fronds	Acute	–	Round	F1, H1		
16) Angle of pinnules against costae	60–70°	–	71–80°	–	81–85°	H1
17) Venation	Free	Except F & G groups	Costal veins triangular	F group	Costal veins areolate	G group
18) Distribution elevation	≤ 1000 m	Except F group	> 1000 m	F group		–
18) Habitats	Full sun, seacoast	A1, A6	Semi–shade, near seacoast		Under forest	–

The alleles corresponding to the character state are difficult to infer.

Plants with homozygous genotypes provided more apparent evidence of character state/genotype associations, than did heterogeneous taxa (Tables 1 and 3). Number of pairs of basiscopic secondary pinnae were useful key characters in this group. In *P. minor* individuals with two or more pairs of basiscopic secondary pinnae or with tripartite laminae possessed alleles A1, A6, and A13. *Pteris latipinna* (D7D7) had the largest pinnae and the fewest lateral pinnae of all taxa; other taxa with wide pinnae had allele D7, including *P. fauriei*, *P. kawabatae*, *P. laurisilvicola*, and *P. natiensis.* It appears that the *P. latipinna* genome is associated with broad pinnae.

The triangular costal veins of *P. arisanensis* are more-or-less intermediate between areolate costal veins, such as in *P. biaurita*, and the free venation of some other species, suggesting a possible hybrid origin of *P. arisanensis*. Homozygous *P. arisanensis* (F7F7F7, Chao2135), however, had triangular costal veins, and homozygous *P. biaurita* (G1G1, Chao3056) had areolate costal veins, thus hybridization does not appear to account for the areolate veins of the former species. By contrast, hybrids (F7G1*, F16G1) between two species exhibit areolate costal veins and were identified as *P. biaurita*.

It is thought that putative hybrids had intermediate phenotypes between two parental taxa but others were more similar to one or the other parent^67^. Hybrids with different genotypes could be morphologically similar if they had the same allele, from the same parental taxon. For example, *P. kawabatae*, *P. kiuschiuensis*, *P. natiensis*, and *P. satsumana* have basal pinna segments connecting to midribs and they share allele C3. Although no sample had a homozygous genotype of C3, the C3 allele was associated with this character. The segments’ angle against midribs in *P. kiuschiuensis* var. *kiuschiuensis* and *P. setulosocostulata* was 80°–85°, wider than all the other taxa, and both taxa had H type alleles. Thus the H allele group appeared to be associated with the larger angle of the segments against midribs.

Some putative hybrids showed ecological intermediacy between parental taxa. For example, *Pteris fauriei* (A1A6D7, A6D7*) occurs in semi-shady environments, whereas the parental taxon *P. minor* (A1A1, A1A13, A1A6, A6A6) grows in open habitats and *P. latipinna* (D7D7) in shady habitats.

Most plants in the *P. fauriei* group are found below 1000 m elevation, except some plants of *P. arisanensis* (D7F4F5, F7F7F7) and *P*. *biaurita* (F7G1*, F16G1) can occur up to 2000 m. The *Knox3* alleles of the F and G groups appeared to be associated with elevational range. Homozygous samples (i.e., putative parents) of *P*. *biaurita* (G1G1) were located in Southeast Asia at low elevation, whereas F group alleles were associated with occurrence at high elevation, above 1000 m. Hybrids (with both F and G group alleles) had broad elevational ranges (*P*. *biaurita* F7G1*, F16G1, low & high; Table 1, Supplementary Table 1).

## Discussion

In recent evolutionary radiations, genetic and morphological divergences are low because of the short evolutionary time scales involved^68–70^. According to Chao, et al.^60^, the main *fauriei* clade arose around 5 Myr, and most taxa diversified around 2 Myr. Our cpDNA tree topology is based on the same markers as that study and supported a recent radiation of the *P. fauriei* group because most taxa were separated by distinctly shorter branches, corresponding to short time periods inferred in the study of Chao, et al.^60^. We revealed a complex hybridization network in the *P. fauriei* group, composed of morphologically similar taxa but also putative hybrid taxa exhibiting morphological intermediacy between probable parental species. This large number of hybrid taxa adds significant morphological complexity to the group leading to difficult taxonomic issues^51^. Furthermore, the evolution of the *P. fauriei* group accounts for a significant amount of the diversity of sect. *Campteria* in *Pteris*, i.e., 18 species, and especially the apogamous elements in Asia^45^. Sect. *Campteria* consisting of more than 60 species, could be the biggest section in *Pteris*^71^ and has more hybridizations, such as in the *P. quadriaurita* complex^41^. Hybridization is a key mechanism facilitating the diversification of sect. *Campteria*.

Among the 18 species of the *P. fauriei* group, some species are phylogenetically distinct from the main clade. We have clarified the hybridization patterns of *P. arisanensis* and/or *P. biaurita*. Although *P. confusa* was thought to be a synonym of *P. arisanensis*^56^, our data suggest that they are different species^51^. We found that *P. confusa* and *P. praetermissa* have the same material lineage. However, because of limited materials, the hybridization involving *P. confusa*^7^ is still unclear.

The assignment of homoeologs is complicated in taxa involved in hybridization and polyploidy^72–74^. Recently, more applicable methods for phasing gene copies into polyploid subgenomes are now in development, focusing on several loci^75^ or target capture data^76^. Even though we found no evidence of possible introgression in the *P. fauriei* group, we revealed that multiple hybridizations contributed to offspring exhibiting diverse phenotypes. Frequent hybridization with low introgression is found in hybrid zones of cacti, apparently due to postzygotic isolation^77^.

Diversification of the *P. fauriei* group appears to be related to different environments. For example, light intensity of *P. fauriei* (apogamous; semi-shady), is an intermediate habitat between its parental taxa *P. minor* (sexual; open) and *P. latipinna* (apogamous; shady). By contrast, homozygous plants of *P. arisanensis* and *P. biaurita* were located at high and low elevation, respectively, and putative hybrids of the two species showed a wider elevational range than the parents, essentially representing the addition of ranges of both parental taxa.

Cases of apparently extinct diploid parental taxa are common in many fern complexes^9,48,49,78^ and in the *P. fauriei* group as well. The distributions of extant parental taxa are much narrower than those of the hybrids, which might imply the decline of parental taxa. For example, *P. latipinna* is an important parent, while the known populations are fewer than ten in China and Taiwan and *P. minor* is limited to Iriomote Isl. (Japan) and Taiwan^51^. Their hybrid *P. fauriei* occurs across a broader area from Japan, Taiwan, and eastern China. *Pteris oshimensis* var. *oshimensis* is distributed across Japan, but the paternal plants are only found in Amami Is, Japan^51,52^. Thus, the ranges of hybrids often exceed those of their parents. The wide distribution of the *P. fauriei* group also suggests that hybridization has enhanced range expansion of the entire group^79^.

After long-distance dispersal, apogamy and gametophytic selfing could be adaptive because either is more likely to produce a new population than would sexual reproduction between gametophytes^80^. Apomictic angiosperms (i.e., asexually reproducing via seeds) tend to predominate in environments unfavorable for sexual reproduction, such as at higher latitudes and elevations, and have a wider distribution than their sexual relatives^81,82^. The apogamous triploid fern *Myriopteris gracilis* has a wide distribution which might be related to its ability to reproduce asexually^83^. Niche differentiation of apogamous and sexual fern taxa has also been documented previously^38,84,85^ similar to what we have observed in the *P. fauriei* group in the current study^58^. However, whether those differences result from reproductive modes and/or higher fitness of certain genotypes needs to be explored further.

Within the *P. fauriei* complex, apogamy is the main reproductive mode with only three sexual taxa. In general, asexual taxa, and lineages with low genetic variation, are thought to be less “adaptable” than are sexual taxa^42^. Furthermore, without recombination, deleterious mutations could accumulate and decrease the fixation of beneficial mutations (Muller’s ratchet)^86,87^. However, new genetic combinations from hybridization could induce phenotypic differentiation of a hybrid from its progenitors^21,88,89^. In the *P. fauriei* group, the extant hybrid taxa, even being apogamous, are apparently the survivors of natural selection and have putatively retained adaptive genotypes that arose from hybridizations, even though some of their parental taxa may be extinct.

Furthermore, apogamous taxa have evolutionary advantages that might overcome the potential deleterious effects of low genetic variation. Epigenetic modification has been proposed to explain high fitness of apogamous taxa^34^. Moreover, Klekowski^90^ proposed that genetic segregation is possible via homoeologous chromosome pairing during sporogenesis leading to genetic variation in apogamous ferns as was found in the study of *Cyrtomium fortunei* (Dryopteridaceae)^91,92^.

Of the seven diploid putative parental taxa, five are apogamous. We hypothesize that some parental taxa with apogamy in the *P. fauriei* group were primarily sexual and then apogamy developed subsequent to the production of hybrids. Apogamous homozygous diploids are more likely derived from sexual diploids rather than from hybrid taxa. The exact mechanisms of sexual taxa giving rise to apogamous taxa could be related to environmental factors such as light, water, and sugar^93–95^. Some critical genes expressed during reproduction could control the process of apogamy^96–100^. Apogamous gametophytes could contribute functional male gametes, which could account for the origin of some triploid taxa in the *P. fauriei* group.

The putative reticulate evolution of the *P. fauriei* group demonstrates a pattern of hybridization in evolutionary radiation, with limited or no introgression. Parental taxa have close phylogenetic relationships and novel genetic combinations in hybrids led to a rapid increase of phenotypic variation, such as diversification of niche fidelities, compared to parental taxa. Apogamy leads to the genetic fixation of some successful hybrid lineages under natural selection. While some parental species could be extinct, some of their genetic diversity will be preserved within the hybrids. Hybridization could lead to abundant taxa, more phenotypic variation, and broader distributions (as discussed above). Our ongoing and future research will explore the relationship of niche differentiation to hybridization, especially by exploring ecophysiological characters and environmental conditions. We hope to know if hybridization plays a role to maintain genetic variation and increase adaptation of ferns.

## Methods

### Sampling

Voucher specimens of samples were deposited at the herbarium of the Taiwan Forestry Research Institute (TAIF; Supplementary Table 1). All sampled plants were identified based on the morphology of type specimens and original protologues; the plants that were morphologically the most similar to the types were marked as representatives of specific species. To identify the plants, morphological characteristics were examined following Chao, et al.^51^. Use of plant material in the study complied with relevant institutional, national, and international guidelines and legislation.

### Ploidy analysis and reproductive systems

Ploidy levels of samples were determined by flow cytometry, if fresh leaves were available, following the methods Chao, et al.^9^. Chromosome numbers of some samples had been counted in previous studies^46,52^. Previously published accounts of ploidy levels and reproductive modes of the *P. fauriei* group were also used (Table 1).

The fact that the sexual *Pteris* plants produce 64 spores per sporangium and apogamous plants produce 32 or fewer spores per sporangium was used to infer the reproductive mode of each plant, corresponding to sexual or apogamous^29,31,59,101^. Five mature sporangia were picked randomly from each plant and spores were counted under a microscope.

### DNA isolation, amplification

Materials for molecular analyses were preserved in silica gel. Total genomic DNA was extracted using a modified cetyltrimethylammonium bromide (CTAB) method^102^. Two plastid gene markers, *rbcL* and *matK*, were amplified by the primers employed in previous *Pteris* studies^57^. The two nuclear DNA (nDNA) putative single-copy genes, *Knox3* and *IBR3*, were analyzed by single-strand conformation polymorphism (SSCP; see description below) and sequenced on the Illumina Miseq platform*.* Five primers for *Knox3* were designed based on transcriptome sequencing data^103^: *Knox3*PF2 (ACA TTC AAG GAG CAG CTT CAG C) and *Knox3*PR3 (CTC TTG CCT AAC GCG CTC CAT G) for the SSCP analysis, *Knox3*PF2NE (CTT CAG CAG CAT GTA CGG GTT CA) and *Knox3*PR3 NGS (CTC TTG CCT AAC GCG CTC CAT G) for the SSCP product amplification, and *Knox3*PF2NE and *Knox3*PR4 (CAT CCT CAT CGT CCG ACA TGG T) for Miseq analyses. We tested the *IBR3* primers *IBR3*.1_4321F2 and *IBR3*.1_4321R2^104^ and modified them for *Pteris* species for the Miseq analyses: primers Pteris.*IBR3*.1_F (CGC ATA TTC ACA GAC CCT) and Pteris.*IBR3*.1_R (GCC AGA TAT TGT TTA GCC CAC C). All primers for Miseq were designed to target sequences shorter than 500 bp. Both forward and reverse primers for *Knox3* and *IBR3* were synthesized with an 8-base barcode to produce amplicons; the barcodes were designed as recommended by Roche^105^.

### Single-strand conformation polymorphism

Some *Knox3* data were analyzed by SSCP. The SSCP analyses were conducted by isolating the nDNA loci from PCR products for each individual through separation on SSCP gels, following the methods of Ebihara, et al.^8^. Individual bands were isolated from gels and purified by the Gel/PCR DNA Fragment Extraction Kit (Geneaid Biotech Ltd. Taipei, Taiwan) and amplified by primers *Knox3*PF2NE and *Knox3*PR3 for further Sanger sequencing.

### Library preparation, sequencing and quality assessment for NGS

Each amplicon was extracted with the illustra GFX PCR DNA and Gel Band Purification Kit (GE, UK). The amplicon mixtures were pooled with equal quantities of DNA, then a total amount of 150 ng of amplicons was used as input material for the DNA library preparations. The sequencing library was generated using the Truseq Nano DNA HT Sample Prep Kit (Illumina, USA) following the manufacturer’s recommendations, and index codes were added to each sample. DNA fragments were ligated with the adapter for Illumina sequencing, followed by further PCR amplification. Then the PCR products were purified (SPRIselect reagent, Beckman) and DNA size spectra were determined using an Agilent 2100 Bioanalyzer and quantified with a Qubit fluorometer (Invitrogen, Carlsbad, California, USA.). Finally, the DNA libraries were sequenced using the Illumina Miseq platform, and 300 bp paired-end reads were generated. Sequencing output was deposited in GenBank (Supplementary Table 1).

### Bioinformatic analysis for nDNA data

Raw data were cleaned by FastQC^106^ and Trimmomatic^107^ to remove the adapter and low quality bases and reads. The forward and reverse reads were merged using PEAR^108^. The cleaned data were demultiplexed and clustered (including chimeras removed), and chimeras were removed using PURC^109^. We kept the clusters comprising the three (for diploid samples) or four highest number of reads (for triploid or ploidy unknown samples). The barcode sequences were identified and removed, that is, each read was assigned to its source accession, then primers were trimmed. The sequences obtained from SSCP and Sanger sequencing were used as the reference sequences for the clustering of consensus sequences. The sequences representing clusters of less than 100 original reads were removed. The consensus sequences of each sample were the alleles of nDNA genes and were ready for downstream phylogenetic analyses. The sequences of the *Knox3* gene, both from SSCP (and Sanger sequencing) and NGS, were pooled together.

### Data analysis

Sequences were automatically aligned using MUSCLE^110^ and then manually edited with BioEdit 7.1.3^111^. Using DnaSp^112^, the sequences of the cpDNA and two nDNA markers were clustered by haplotypes and type of alleles, respectively. To exclude errors in amplicon data, we only kept the alleles found in two or more samples. We hypothesized that the taxa that had only one allele type were homozygous and classified them as parental taxa (non-hybrid taxa), regardless of whether their reproduction modes were sexual or apogamous. Allele numbers of genotypes were supported by ploidy data. When the allele number was smaller than the ploidy level, it was difficult to infer the exact allelic composition; in those cases, “*” stands for unassorted alleles^8,9^.

Maximum likelihood analyses were performed for cpDNA haplotypes and nDNA sequences using the program GARLI v.2.0.1019^113^. *Pteris* species in sect. *Campteris* were included, and species in other sections, including *P. bella* Tagawa, *P. formosana* Baker, *P. kidoi* Sa.Kurata, *P. longipes* D.Don, and *P. wallichiana* J.Agardh, were chosen as outgroups^60,71^. For the three sequence datasets—cpDNA, *Knox 3*, and *IBR3*—three phylogenetic analyses were conducted by ten independent runs, from different random sequence-order starting trees, based on automatic termination following 10,000 generations without a significant topological change. The ML bootstrap support for each clade was assessed by performing 1000 bootstrap replicates, each replicate with one single tree search with the same search parameters as above. A 50% majority rule consensus tree was then calculated using PAUP* v. 4.0b10^114^. Gaps were treated as missing data. Then the nDNA genotypes, *Knox3* and *IBR3*, were compared visually to the cpDNA markers to infer possible hybridization patterns. A phylogenetic network was visualized with Dendroscope 3^115^.

We also used homologizer^75^ to phase the alleles of the two nDNA genes (linking the tree of the two genes) and infer a phylogeny. The potential parental taxa with homozygous nDNA genotypes were fixed firstly to infer the pattern of the subgenome (or allele) evolution of this group. The two genes were modelled by an independent GTR substitution model and exponential priors (mean = 0.1) on branch lengths. The analysis was based on a Bayesian Markov chain Monte Carlo (MCMC) approach for four runs; the run with highest posterior (mean) was selected. Trees were run for 15,000 generations and the first 0.1 trees were discarded as burn-in. We assessed convergence by Tracer v.1.7^116^ (ESS over 200) and summarized the trees into a single tree and rooted it by FigTree^117^. Then the phasing estimates were plotted by RevGadgets^118^.

## Supplementary Information

Supplementary Information.
